# Spleen assessment after laparoscopic transperitoneal left adrenalectomy: preliminary results

**DOI:** 10.1007/s00464-015-4363-y

**Published:** 2015-07-03

**Authors:** Pasquale Cianci, Alberto Fersini, Nicola Tartaglia, Amedeo Altamura, Vincenzo Lizzi, Luca Pio Stoppino, Luca Macarini, Antonio Ambrosi, Vincenzo Neri

**Affiliations:** Department of Medical and Surgical Sciences, University of Foggia, Luigi Pinto Str 1, 71122 Foggia, Italy

**Keywords:** Laparoscopic adrenalectomy, Wandering spleen, Endocrine surgery, Minimally invasive surgery, Adrenal diseases

## Abstract

**Background:**

Several laparoscopic approaches to the adrenal gland have been described. We prefer the lateral transabdominal approach. The aim of this study is to evaluate prospectively the presence of any anatomical and dynamic changes in the spleen after laparoscopic transperitoneal left adrenalectomy (LTLA), which can cause an increased risk of early and late complications.

**Methods:**

We have evaluated 21 patients before and 6 months after surgery in order to verify the possible presence of a wandering spleen. A clinical and instrumental follow-up [ultrasound (US), magnetic resonance (MR)] were performed. During US protocol, in supine, right lateral, and orthostatic position, the longitudinal and anteroposterior diameter of the spleen and the resistive index within 3 cm of the origin of the splenic artery in three different measurements averaged were measured. MR protocol evaluated, in supine and right lateral position, the splenic volume and its distances from the diaphragm dome and the lateral margin of the costal arch.

**Results:**

*p* Values calculated for each parameter were not statistically significant. Our results confirm the absence of any anatomical and dynamic changes in the spleen after LTLA.

**Conclusions:**

The most common complications after laparoscopic adrenalectomy are well known and widely described. Our experience does not exclude the occurrence of a wandering spleen, but allows us to state that a rightful mobilization of the pancreaticosplenic block can avoid this event, and in agreement with other authors, the presence of a wandering spleen remains an isolated complication.

The laparoscopic approach for adrenalectomy was first described by Gagner [1]. Since its introduction, the technique has rapidly gained popularity among surgeons dealing with adrenal disease, and it is presently considered the “gold standard” procedure for the removal of benign and selected malignant adrenal pathologic masses even greater than 7 cm [2, 3].

The most accepted route to the adrenal gland is the laparoscopic transperitoneal approach, with the patient placed on hyperextended lateral flank [3, 4]. On the left side, the pancreatic tail and the spleen are fully mobilized and dropped medially. A greater mobilization of the pancreaticosplenic would have recreated the conditions of a wandering spleen manifested by its complication such as gastric volvulus [5–9]. The overall complication rate associated with laparoscopic adrenalectomy was 9.5 % (range 2.9–20) [3]. The most common complications after laparoscopic adrenalectomy are well known and widely described include bleeding, organ injury, wound hematoma and wound infection, systemic complication [10, 11]. The aim of this study is to evaluate prospectively the presence of any anatomical and dynamic changes in the spleen after laparoscopic transperitoneal left adrenalectomy (LTLA), which can cause an increased risk of early and late complications.

## Materials and methods

From January 2008 to July 2013, we treated 71 patients with adrenal diseases in our Department. During the same period, 43 laparoscopic procedures were performed and of these we evaluated only the 21 laparoscopic left adrenalectomies, 9 males (42.86 %) and 12 females (57.14 %) with a mean age of 52.05 years (range 17–73).The characteristics of the patients are shown in Table 1. Each case has been studied by our team of endocrinologists prior to intervention, and the indications for surgery were nonfunctioning adenoma (*n* = 6), Conn’s syndrome (*n* = 4), pheochromocytoma (*n* = 6), nodular cortical hyperplasia (*n* = 4), and metastatic lung cancer (*n* = 1) (Table 2). The exclusion criteria were bilateral adrenalectomy, previous abdominal surgery, and concomitant surgical diseases. The laparoscopic operation was performed in all instances using a transperitoneal lateral approach with the patient placed in an almost full lateral position and with the operating table broken to maximum extension to allow the space between rib cage and pelvis to open fully. After access and placement of three trocars, the first step was to incise the lienal attachments to the diaphragm along the lateral border of the spleen at the splenorenal ligament, the dissection is closed when the short gastric vessels are identified posteriorly. The spleen then falls medially under gravity. The mobilization of the splenic flexure was performed only when absolutely necessary. After informed consent we have evaluated the spleen with color Doppler ultrasound and MRI scan before and 6 months after surgery. During ultrasound (US) protocol using curvilinear variable-frequency transducer with the patients in different position (supine, right lateral, orthostatic), the longitudinal and anteroposterior diameter of the spleen and the resistive index within 3 cm of the origin of the splenic artery in three different measurements averaged were measured. The same radiological team performed magnetic resonance (MR) protocol using a 1.5-T unit. In supine and right lateral position were measured the splenic volume and its distances from the diaphragm dome and the lateral margin of the costal arch. Data are presented as mean values and analyzed using a two-tailed *t* test for paired data (Microsoft Excel 2000, Microsoft, Redmond, WA, USA). Statistical significance was taken to be a *p* value less than 0.05.

**Table 1:** Demographic characteristics of the patients

Sex	
Male	9 (42.86 %)
Female	12 (57.14 %)
Age	
Mean	52.05
Range	17–73
BMI (Kg/m^2^)	
Mean	24.62
Range	19–30
A.S.A. score	
I	11/21 (52.40 %)
II	7/21 (33.30 %)
III	3/21 (14.30 %)
IV	0/21 (0 %)
V	0/21 (0 %)

**Table 2:** Indications for surgery

Nonfunctioning adenoma	6/21 (28.57 %)
Conn’s syndrome	4/21 (19.05 %)
Pheochromocytoma	6/21 (28.57 %)
Nodular cortical hyperplasia	4/21 (19.05 %)
Metastasis (lung cancer)	1/21 (4.76 %)

## Results

A total of 21 LTLA were followed during the study period. Intraoperative and postoperative outcomes are summarized in Table 3. The average operation length was 159 min (range 120–220), and no bilateral lesion was included. Mean size of the lesions was 2.73 cm (range 0.7–7). The average blood loss was 77.25 ml (range 50–110), and no patients required transfusion. The conversion rate was 0 %, and the complication rate was 14.28 %. This last includes one case of hemoperitoneum in the immediate postoperative period, caused by bleeding from a small branch of the adrenal vein promptly resolved surgically. Postoperative courses were free of complications in 19 patients. In the remaining two patients, there was one case of atrial fibrillation and one wound infection. No mortality occurred.

**Table 3:** Intraoperative and postoperative data of patients undergoing LTLA

Operative time (min)	
Mean (SD)	159 (36.49)
Median	150
Range	120–220
Blood loss (ml)	
Mean (SD)	77.25 (16.66)
Median	77.5
Range	50–110
Tumor size (cm)	
Mean (SD)	2.73 (1.53)
Median	2.5
Range	0.7–7
Conversion rate (%)	0
Postoperative ambulation (hours)	
Mean (SD)	31.25 (9.10)
Median	27.5
Range	22–50
Postoperative hospitalization (days)	
Mean (SD)	4.05 (1.05)
Median	4
Range	3–6
Perioperative complications rate (%)	4.75
Postoperative complications rate (%)	9.50

During the US protocol, no significant difference in the pre- and postoperative longitudinal diameter of the spleen was observed (*p* = 0.531). Neither anteroposterior diameter of the spleen (*p* = 0.394) nor the resistive index (*p* = 0.454) was significantly different in these reports. Also MR outcomes showed no difference between pre- and postoperative averages of splenic volume (*p* = 0.849), distance from its lateral margin to costal arch (*p* = 0.428), and its distance from diaphragm dome (*p* = 0.080). These results confirm the absence of any anatomical and dynamic changes in the spleen after LTLA (Table 4).

**Table 4:** US and MR protocol findings before and six months after surgery

Parameters	Protocol	Preoperative findings (mean)	Postoperative findings (mean)	*p* value (<0.05)
Splenic volume (cm^3^)	MR	212.2476	210.2857	0.849
Spleen–lateral margin/costal arch (distance in cm)	MR	0.64	0.680952	0.428
Spleen–diaphragm dome (distance in cm)	MR	0.62	0.7	0.080
Longitudinal diameter of the spleen (cm)	US	10.92	10.7	0.531
Anteroposterior diameter of the spleen (cm)	US	4.9	4.75	0.394
Resistive index	US	0.612381	0.626	0.454

## Discussion

Since its introduction in 1992 [1], LA has rapidly became the procedure of choice for the surgical management of most adrenal tumors. Comparing to open adrenalectomy, laparoscopic adrenalectomy offers better clinical outcomes, lower less perioperative morbidity, shorter hospitalization, and better cosmetic results [3, 12–16]. Transabdominal and retroperitoneal approaches are most common [17]. The majority of surgeons perform the lateral transperitoneal access that favors excellent exposure and allows gravity to aid in the retraction of adjacent organs. The posterior retroperitoneal approach is an alternative that avoids the peritoneal cavity without mobilization of pancreas and spleen and may be helpful for patients with abdominal adhesions or patients requiring bilateral adrenalectomies; however, exposure and working space are limited, and anatomical relationship may not be as familiar. The third and less common access is an anterior transperitoneal approach, which affords the traditional view of anatomy but requires considerable effort to retract adjacent organs and maintain exposure [18]. We prefer the first way that requires less dissection, better retraction of the adjacent organs, and easy removal of the surgical specimen. We used the precocious binding of the adrenal vein, the greatest care in hemostasis and in dissection, preserving the anatomical integrity of the gland and the positioning of a tubular drainage at the end of the operation [19]. The most common complications after laparoscopic adrenalectomy are well known and widely described [10, 11]. We have evaluated 21 patients before and 6 months after surgery in order to verify the possible presence of a wandering spleen. In addition to a clinical and ultrasonographic evaluation, we have used also a MR protocol. The literature suggests that Doppler ultrasound is used to check the spleen position and parenchymal flow [20–23]. Some authors describe CT scan as the best choice to identify this rare condition [24], although Buckley et al. [25] suggest that MR imaging may confer certain advantages because of its lack of ionizing radiation, as well as its superior tissue characterization, and it is not operator dependent. Our preliminary results confirm the absence of any anatomical and dynamic changes in the spleen after LTLA, and we have compared them to the largest series in the literature (Table 5). The authors reported were chosen based on the following criteria: (1) LTA as surgical approach, (2) three trocars used, (3) single institution experience, and (4) only adult patients evaluated.

**Table 5:** Outcomes of the laparoscopic transperitoneal adrenalectomy (LTA) in selected large series

References	No. of procedures	Operative time (min)	Conversion rate (%)	Complication rate (%)/No. of WS	Postoperative hospitalization (days)	Mortality (%)
Henry et al. [10]^a^	169	129	5	7.5/0	5.4	0
Guazzoni et al. [26]^a^	161	160	2.5	5.1/0	2.8	0
Porpiglia et al. [27]^a^	125	139	3.2	11.2/0	4	0.8
Gagner et al. [28]^a^	100	123	3	12/0	2.4	0
Conzo et al. [29]^a^	88	137.33	1.13	5.6/0	3.5	0
Piccoli et al. [30]^b^	24	89	0	8/0	2	0
Neri et al. [19]^b^	21	159	0	14.28/0	4.05	0

*WS* wandering spleen

^a^Data include laparoscopic right and left adrenalectomies, ^b^ Data include only laparoscopic left adrenalectomies

The unique case of alteration of the spleen assessment after LTLA was described by Corcione et al. [5]. He observed a case of a 57-year-old woman who has undergone LTLA and in the third postoperative month, had an acute gastric volvulus requiring surgical open treatment. In his opinion, a probable gastrosplenic ligament laxity and an excessive mobilization of the pancreaticosplenic block can be the etiology of this singular complication. In the wake of this case, Piccoli et al. [30] have evaluated 24 patients with a clinical and ultrasonographic follow-up that showed no evidence of postoperative wandering spleen. Following the procedure described by Gagner [28], we begin the mobilization of the spleen dissecting the lienal attachment to the diaphragm along the lateral border at the lienorenal ligament. Superiorly the dissection is stopped when the short gastric vessels are identified posteriorly. After this, the spleen falls medially and this favors the complete exposure of the adrenal gland. Our experience does not exclude the occurrence of this event, but allows us to state that a rightful mobilization of the pancreaticosplenic block can avoid the occurrence of a wandering spleen, and in agreement with other authors, the presence of a wandering spleen remains an isolated complication.
